# Longitudinal biomarker trajectories accompanying trichoscopic change after PRP-based regimens for alopecia: a complementary multi-marker analysis of a real-world cohort

**DOI:** 10.25122/jml-2026-0024

**Published:** 2026-02

**Authors:** Adelina Vrapcea, Dumitru Rădulescu, Cristina Violeta Tutunaru, Sandra Alice Buteica, Elena Stănciulescu, Cătălina Gabriela Pisoschi

**Affiliations:** 1 Doctoral School, University of Medicine and Pharmacy of Craiova, Craiova, Romania; 2 Department of Surgery, University of Medicine and Pharmacy of Craiova, Craiova, Romania; 3 Department of Dermatology, University of Medicine and Pharmacy of Craiova, Craiova, Romania; 4 Pharmaceutical Chemistry Department, University of Medicine and Pharmacy of Craiova, Craiova, Romania; 5 Department of Biochemistry, University of Medicine and Pharmacy of Craiova, Craiova, Romania

**Keywords:** platelet-rich plasma, nonscarring alopecia, trichoscopy, longitudinal analysis, biomarker trajectories, inflammatory indices, VEGF, TNF-α, vitamin D

## Abstract

Platelet-rich plasma (PRP) is widely used in nonscarring alopecia, yet the biological context accompanying measurable trichoscopic change in routine practice remains incompletely described. This study aimed to provide a complementary longitudinal analysis of the same real-world treatment cohort, focusing on within-subject trichoscopic change and paired biomarker trajectories rather than on baseline response stratification. In this ambispective observational cohort, participants underwent paired baseline (T1) and follow-up (T2) assessments after PRP, PRP + exosomes, PRP + nutricomplex, or nutricomplex alone. Total hair density (hairs/cm^2^) was summarized as absolute change (Δ) and percent change (%Δ). Longitudinal systemic profiling included complete blood count (CBC)-derived inflammatory indices—neutrophil-to-lymphocyte ratio (NLR), derived neutrophil-to-lymphocyte ratio (dNLR), systemic immune-inflammation index (SII), platelet-to-lymphocyte ratio (PLR), and systemic inflammation response index (SIRI)—as well as selected soluble markers, including vitamin D, tumor necrosis factor-alpha (TNF-α), vascular endothelial growth factor (VEGF), and transforming growth factor-beta 1 (TGF-β1). Paired changes were evaluated using Wilcoxon signed-rank tests, between-regimen differences in %Δ density with Kruskal–Wallis testing, and biomarker-change associations with Spearman correlation and FDR control. Among 129 participants, total hair density increased from T1 to T2, although the magnitude of improvement varied substantially across individuals. Regimen-level %Δ density showed numerical dispersion without statistically conclusive between-group separation. CBC-derived indices and soluble markers also displayed heterogeneous paired trajectories, and exploratory correlations suggested that selected biomarker change-scores tracked with %Δ density. This manuscript extends the cohort-level narrative by showing that early trichoscopic improvement under PRP-based care is accompanied by heterogeneous longitudinal biomarker modulation. Framed as a companion, trajectory-oriented analysis, the study supports interpreting response heterogeneity through paired clinical-biomarker dynamics rather than through a single summary endpoint alone.

## INTRODUCTION

Hair loss is a frequent reason for dermatology consultation and can substantially affect quality of life. Beyond clinical inspection, objective quantification of treatment response has become increasingly important, particularly in androgenetic alopecia and other nonscarring alopecias, where change can be gradual and heterogeneous. Computer-assisted trichoscopy/phototrichogram methods (e.g., TrichoScan) enable reproducible measurement of hair density and related parameters and have been widely used to monitor therapeutic effects over time [1].

Among regenerative approaches, autologous platelet-rich plasma (PRP) has gained popularity as an adjunct or alternative treatment for androgenetic alopecia. The rationale for PRP is based on platelet activation and release of bioactive mediators that can influence follicular cycling, perifollicular microenvironment, and tissue remodeling [2]. Randomized controlled data have supported improvements in hair density compared with placebo in androgenetic alopecia, although protocols (preparation, activation, injection technique, number of sessions, and follow-up intervals) vary considerably [3]. This variability complicates synthesis across studies and contributes to mixed estimates of efficacy across systematic reviews and meta-analyses. [4,5]. Contemporary reviews emphasize both the clinical signal in favor of PRP and the need for standardized protocols and for more mechanistic, biomarker-informed studies [6].

Mechanistically, PRP is enriched with platelet-derived growth factors and cytokines (e.g., vascular endothelial growth factor (VEGF), transforming growth factor-beta (TGF-β), and others) that are involved in angiogenesis, extracellular matrix remodeling, and cell signaling relevant to tissue repair [7,8]. These mediators are biologically plausible contributors to hair follicle support and to the shift toward anagen maintenance reported in some clinical studies [6–8]. However, clinical response is not uniform. A key challenge is that trichoscopic improvement occurs in some individuals, while others show minimal change, suggesting that host biology and the systemic milieu may modulate the response.

In parallel with PRP’s local effects, increasing attention has been paid to systemic inflammatory activity in nonscarring alopecias. Hematologic ratios and composite indices derived from complete blood count (CBC)—such as the neutrophil-to-lymphocyte ratio (NLR), derived NLR (dNLR), systemic immune-inflammation index (SII), platelet-to-lymphocyte ratio (PLR), and systemic inflammation response index (SIRI)—have been explored across inflammatory and immune-mediated conditions, including alopecia areata and related inflammatory hair-loss contexts [9–11]. These indices are attractive because they are low-cost, widely available, and interpretable as proxy measures of systemic inflammatory balance. Standard formulas for calculating these indices from neutrophil, lymphocyte, platelet, and monocyte counts are established in the literature [12]. In addition, soluble markers such as TNF-α and growth factors (e.g., VEGF, TGF-β1) provide complementary insights into inflammatory signaling and regenerative pathways that may be relevant to hair follicle cycling. [7,8].

Vitamin D has also been frequently discussed in the context of nonscarring and scarring alopecias, with meta-analytic evidence indicating an association between vitamin D deficiency and several alopecia disorders [13]. Because vitamin D influences immune regulation and keratinocyte biology, its longitudinal changes may be informative when evaluating systemic status in patients undergoing combined or adjunctive interventions.

Despite accumulating literature, relatively few studies integrate:

- objective trichoscopic outcomes;
- longitudinal pre–post biomarker trajectories;
- real-world PRP-based regimens (including combinations such as PRP with exosome-based products or nutraceutical complexes) within the same analytic framework.

This gap is relevant because routine care often involves individualized multimodal regimens rather than uniform protocols, and because the biological correlates of improvement may be better understood as evolving trajectories than as isolated baseline predictors.

Therefore, the present study aimed to quantify trichoscopic response from baseline (T1) to follow-up (T2) in a real-world cohort treated with PRP-based regimens and to characterize concurrent systemic biomarker trajectories using a panel approach. In the overall doctoral framework, this article is intended as a complementary follow-on analysis using distinct analytic endpoints: whereas related work from the same cohort addresses pre-treatment stratification, the present manuscript focuses on paired longitudinal change, regimen-level patterning, and correlations between biomarker deltas and trichoscopic improvement.

## MATERIAL AND METHODS

### Study design, setting, and timeline

This work was designed as an ambispective observational cohort study conducted in a real-world outpatient dermatology setting. Routinely collected clinical records were used retrospectively, and follow-up evaluations were collected prospectively under an approved protocol. The study period covered October 2024 to October 2025. Participants were evaluated at two standardized timepoints: baseline (T1) and the first available post-treatment reassessment (T2), typically obtained approximately 4 months after completion of the treatment course.

### Participants and analytic cohort

Clinical records were screened for eligibility. Adults evaluated for nonscarring alopecia were included if they had:

- paired trichoscopic measurements at T1 and T2,
- laboratory data sufficient to compute CBC-derived inflammatory indices and to analyze the soluble biomarker panel. Exclusion criteria included lack of treatment exposure, missing paired trichoscopy, incomplete baseline CBC differential data required for index computation, documented autoimmune disease, and evidence of baseline systemic inflammation as recorded in the medical file. After screening, the final analytic cohort comprised 129 participants.

### Treatment regimens and exposure characterization

Treatment allocation reflected routine clinical decision-making and was not randomized. Participants were categorized into four regimen groups recorded as “*schema tratament”*:

- PRP
- PRP + Exosomes
- PRP + Nutricomplex
- Nutricomplex

The number of treatment sessions was recorded as “*nr_sedinte_tratament”* and used descriptively.

### PRP preparation and administration

PRP was prepared from autologous peripheral blood using a closed PRP system (REGEN Lab, Switzerland) with PRP Newlife ACS tubes (10 mL). Samples were centrifuged according to the routine protocol (7 minutes at 4000 rpm). The platelet-rich plasma was used as prepared for intradermal administration.

Scalp injections were performed with 32-gauge needles at approximately 2-4 mm depth, with a total volume of 5-6 mL distributed across the affected scalp regions based on surface area and clinical severity. Because the present article is longitudinal rather than protocol-comparative in emphasis, procedural details are summarized here only to define exposure context.

### Adjunct protocols and microneedling delivery (combined-regimen groups)

Adjunct products (exosome-containing preparation or nutrient complex) were applied only within the corresponding combined-regimen groups. Delivery used a microneedling pen to facilitate transdermal administration, with depth individualized to scalp characteristics (typically 0.5-1.5 mm) and with the practical goal of uniform erythema and minimal pinpoint bleeding.

### Trichoscopy acquisition and outcomes

Trichoscopy was performed at T1 and T2 using a FotoFinder ATBM system.

Four standardized scalp regions were assessed - frontal, bitemporal, vertex, and parietal - under consistent acquisition conditions across visits.

For this companion manuscript, the primary clinical endpoint was total hair density (hairs/cm^2^), summarized as both absolute change (Δ = T2 - T1) and percent change (%Δ = ((T2 - T1) / T1) × 100). This framing was chosen to emphasize longitudinal within-subject evolution rather than baseline response classification:

**Absolute change (Δ):**

Δ = T2 − T1

**Percent change (%Δ):**

%Δ = ((T2 − T1) / T1) × 100

### Systemic biomarker panel and derived inflammatory indices

Laboratory values were extracted from routine testing performed at baseline and, when available, at follow-up, under standard laboratory conditions.

CBC-derived inflammatory indices were evaluated at T1 and T2, including:

- NLR (neutrophils/lymphocytes)
- dNLR (neutrophils/(100 − neutrophils))
- SII (platelets × neutrophils/lymphocytes)
- PLR* (platelets/lymphocytes)
- SIRI* ((neutrophils × monocytes)/lymphocytes)

In addition, the soluble biomarker panel included vitamin D, TNF-α, VEGF, and TGF-β1, analyzed longitudinally as paired values when available. For each biomarker, within-subject change scores were defined as Δ = T2 − T1 (and as percent change when relevant).

### Covariates and coded variables

Demographic and baseline characteristics were collected from structured clinical intake and standardized questionnaires. In this article, these variables are reported primarily to contextualize regimen composition and phenotype mix rather than to construct a baseline prediction model.

### Statistical analysis

Inferential analyses were performed using IBM SPSS Statistics for Windows, Version 26.0 (IBM Corp., Armonk, NY, USA). Figure generation, table exports, and data-wrangling steps used to create publication-ready visualizations were performed using Python 3.13.1. Given skewed distributions and potential outliers in several biomarkers and outcomes, a nonparametric framework was used throughout. Continuous variables are reported as median [IQR], and categorical variables as *n* (%). Paired T1-T2 comparisons were conducted using the Wilcoxon signed-rank test; between-regimen comparisons of %Δ total hair density used the Kruskal-Wallis test; and associations between %Δ total density and biomarker change-scores (Δ) were evaluated using Spearman’s rank correlation. False discovery rate (FDR) adjustment (Benjamini-Hochberg) was applied across biomarker panels, and analyses used pairwise complete cases for each endpoint.

## RESULTS

A total of 129 participants completed paired assessments at baseline (T1) and follow-up (T2), including PRP (*n* = 54), PRP + exosomes (*n* = 33), PRP + nutricomplex (*n* = 24), and nutricomplex alone (*n* = 18). Core baseline context by regimen is summarized in Table 1.

**Table 1:** Core baseline context of the complementary longitudinal cohort, summarized by treatment regimen

Regimen	*n*	Age (years)	No. treatment sessions	Baseline total density (hairs/cm^2^)	Sex
Nutricomplex	18	37.00 [32.25, 44.25]	4.00 [3.25, 4.00]	102.39 [96.05, 121.84]	Male: 16 (88.9%); Female: 2 (11.1%)
PRP	54	37.00 [28.25, 44.00]	4.00 [4.00, 4.00]	103.06 [85.54, 117.79]	Male: 33 (61.1%); Female: 21 (38.9%)
PRP + Exosomes	33	37.00 [30.00, 49.00]	4.00 [4.00, 4.00]	104.25 [94.02, 122.96]	Male: 21 (63.6%); Female: 12 (36.4%)
PRP + Nutricomplex	24	39.50 [36.75, 46.00]	4.00 [4.00, 4.00]	105.44 [100.52, 118.99]	Male: 17 (70.8%); Female: 7 (29.2%)

Continuous variables are reported as median [IQR]. Categorical variables are reported as *n* (%).

Across regimens, median age was broadly similar (approximately 37–39.5 years), the number of treatment sessions clustered around four sessions, and baseline total hair density showed overlapping distributions, supporting the use of this table primarily as a descriptive cohort context for the subsequent longitudinal analyses.

**Figure 1:**
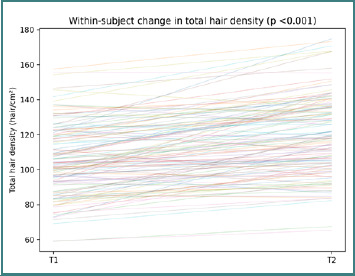
Participant-level paired trichoscopic trajectories in total hair density across the full complementary longitudinal cohort. Each line represents one participant; the title reports the paired Wilcoxon *P* value.

At the cohort level, paired trichoscopic reassessment showed an overall increase in total hair density, providing the clinical context for the accompanying biomarker analyses. Baseline total hair density was 103.93 hair/cm^2^ [91.96, 119.2] and increased at follow-up to 118.23 hair/cm^2^ [102.69, 135.71], with a significant paired shift on Wilcoxon testing (*P* < 0.001). Individual trajectories indicated that most participants improved, while a smaller subset showed limited change or slight decreases (Figure 1).

To express improvement in a scale-free manner, the percent change in total density (%Δ) was computed relative to baseline. At the cohort level, %Δ total density was 11.95% [4.29, 21.79]. Paired changes for total density and systemic biomarkers are summarized in Table 2 using median values, median paired differences with bootstrap confidence intervals, and nonparametric *P* values with FDR correction across biomarkers.

**Table 2:** Paired changes from T1 to T2 for trichoscopic density, CBC-derived inflammatory indices, and soluble markers

Marker	*N* paired	T1 median [IQR]	T2 median [IQR]	Median paired Δ (T2–T1)	Δ 95% CI	*P*	q (FDR)
TNF-α	129	90.08 [56.74, 116.71]	72.40 [47.19, 105.59]	1.58	[0.14, 3.46]	0.014	0.014
NLR	129	4.03 [1.92, 5.10]	3.32 [1.31, 4.26]	-0.62	[-0.69, -0.57]	<0.001	<0.001
PLR*	129	1757.73 [781.98, 2217.65]	1495.05 [636.36, 1939.23]	-182.79	[-225.16, -163.64]	<0.001	<0.001
SII	129	1288.42 [454.04, 1676.80]	1056.07 [308.17, 1415.86]	-175.14	[-204.65, -155.14]	<0.001	<0.001
SIRI*	129	16.90 [10.78, 29.47]	16.52 [8.11, 25.52]	-3.16	[-4.67, -1.43]	<0.001	<0.001
TGF-β1	129	691.27 [621.10, 752.83]	745.55 [651.00, 813.07]	51.91	[34.05, 64.14]	<0.001	<0.001
Total density (hair/cm^2^)	129	103.93 [91.96, 119.20]	118.23 [102.69, 135.71]	12.22	[9.12, 16.37]	<0.001	<0.001
VEGF	129	791.01 [765.97, 810.37]	882.01 [827.82, 967.33]	95.04	[84.93, 116.83]	<0.001	<0.001
Vitamin D	129	27.17 [22.38, 33.59]	31.80 [25.55, 40.96]	3.60	[3.06, 4.32]	<0.001	<0.001
dNLR	129	2.60 [1.48, 3.22]	2.23 [1.05, 2.82]	-0.38	[-0.40, -0.33]	<0.001	<0.001

Values are shown as median [IQR] at each time point; Δ is the median paired difference with a bootstrap 95% CI. *P* values are from paired Wilcoxon tests; q-values are FDR-adjusted. Because Δ represents the median of within-subject paired differences, its sign may differ from the arithmetic difference between marginal medians.

**Figure 2:**
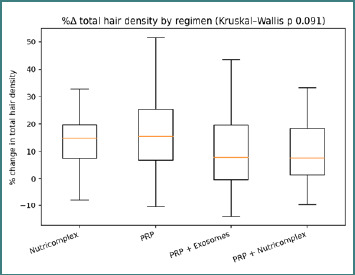
Distribution of percent change (%Δ) in total hair density by regimen. Boxes represent the median and IQR; whiskers indicate the non-outlier data range; the title reports the overall Kruskal-Wallis *P* value.

When stratified by regimen, the distribution of %Δ total density differed across treatment groups (overall Kruskal–Wallis *P* = 0.091). The boxplots illustrate both the central tendency and the within-regimen spread of responses (Figure 2), supporting a cautious interpretation consistent with the reviewers’ request to foreground the exploratory character of regimen contrasts. Regimen-specific medians and IQRs are provided for descriptive comparison (Table 3).

**Table 3:** Descriptive summary of %Δ total hair density by regimen; not intended for causal regimen ranking

Regimen	%Δ total density median [IQR]	Kruskal–Wallis p (overall)
NUTRICOMPLEX	14.91 [7.58, 19.78]	0.091
PRP	15.54 [6.78, 25.46]	0.091
PRP+EXOZOMES	7.87 [-0.28, 19.63]	0.091
PRP+NUTRICOMPLEX	7.70 [1.39, 18.37]	0.091

EBV=Epstein-Barr virus; BCL2=B-cell lymphoma 2; PD 1=Programmed cell death Protein 1; Mfg=macrophages; EGFR=epidermal growth factor receptor

Systemic inflammatory status was evaluated using CBC-derived indices (NLR, dNLR, SII, PLR, and SIRI) together with soluble markers (vitamin D, TNF-α, VEGF, and TGF-β1). Rather than treating these markers as interchangeable proxies, the present analysis tracks their paired movement over time to describe the biological heterogeneity accompanying trichoscopic change.

**Figure 3:**
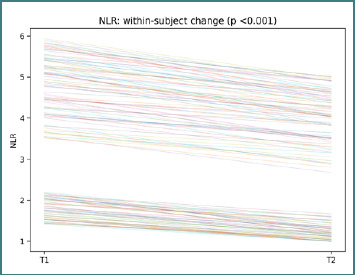
Participant-level NLR trajectories from T1 to T2. Each line represents one participant; the title reports the paired Wilcoxon *P* value.

NLR trajectories show participant-level changes between T1 and T2, with a paired Wilcoxon *P* < 0.001 (Figure 3).

**Figure 4:**
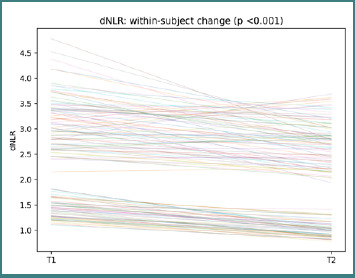
Participant-level dNLR trajectories from T1 to T2. Each line represents one participant; the title reports the paired Wilcoxon *P* value.

The variability in line slopes indicates heterogeneous inflammatory shifts were not uniform across participants, reinforcing the rationale for a trajectory-based interpretation. dNLR trajectories show participant-level changes between T1 and T2, with a paired Wilcoxon *P* <0.001 (Figure 4).

**Figure 5:**
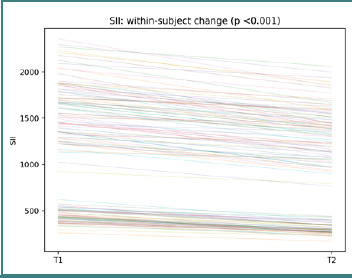
Participant-level SII trajectories from T1 to T2. Each line represents one participant; the title reports the paired Wilcoxon *P* value.

Again, slope heterogeneity suggests that paired inflammatory change is individualized rather than homogeneous at the cohort level. SII trajectories show participant-level changes between T1 and T2, with a paired Wilcoxon test, *P* < 0.001 (Figure 5).

**Figure 6:**
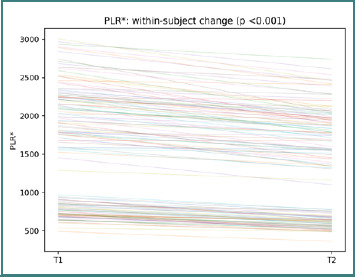
Participant-level PLR* trajectories from T1 to T2. Each line represents one participant; the title reports the paired Wilcoxon *P* value.

This spread of trajectories further supports the interpretation of systemic modulation as heterogeneous across the cohort. PLR* trajectories show participant-level changes between T1 and T2, with a paired Wilcoxon *P* <0.001 (Figure 6).

**Figure 7:**
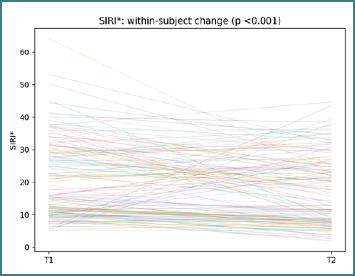
Participant-level SIRI* trajectories from T1 to T2. Each line represents one participant; the title reports the paired Wilcoxon *P* value.

As for the other CBC-derived indices, the direction and amplitude of change varied across individuals. SIRI trajectories show participant-level changes between T1 and T2, with a paired Wilcoxon test, *P* < 0.001 (Figure 7).

These paired profiles indicate that the inflammatory context accompanying clinical improvement is not monolithic.

Soluble markers provided complementary information on systemic signaling. VEGF was included as a growth factor marker, TNF-α as an inflammatory cytokine, and vitamin D as an immunomodulatory factor. Participant-level paired trajectories show that changes vary widely in both direction and magnitude, highlighting substantial inter-individual heterogeneity.

**Figure 8:**
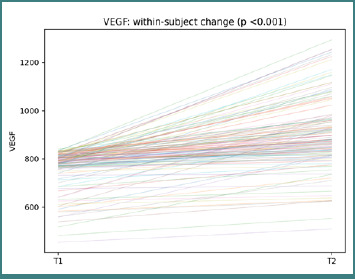
Participant-level VEGF trajectories from T1 to T2. Each line represents one participant; the title reports the paired Wilcoxon *P* value.

In VEGF, participant-level changes between T1 and T2 were observed (paired Wilcoxon *P* <0.001; Figure 8).

**Figure 9:**
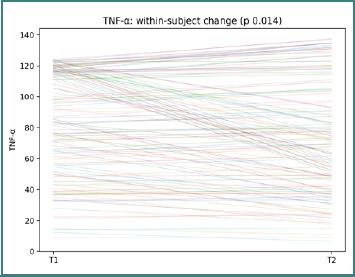
Participant-level TNF-α trajectories from T1 to T2. Each line represents one participant; the title reports the paired Wilcoxon *P* value.

The widespread line slopes highlight marked between-patient variability in growth-factor dynamics. In TNF-α, participant-level changes between T1 and T2 were observed (paired Wilcoxon *P* = 0.014; Figure 9).

**Figure 10:**
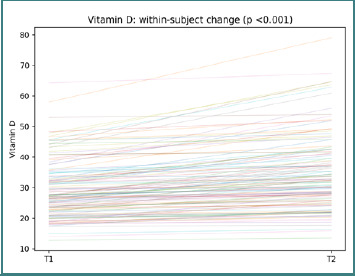
Participant-level vitamin D trajectories from T1 to T2. Each line represents one participant; the title reports the paired Wilcoxon *P*-value.

This pattern again supports a cautious interpretation centered on heterogeneous paired shifts rather than a uniform cohort effect. In vitamin D, participant-level changes between T1 and T2 were observed (paired Wilcoxon *P* < 0.001; Figure 10).

**Figure 11:**
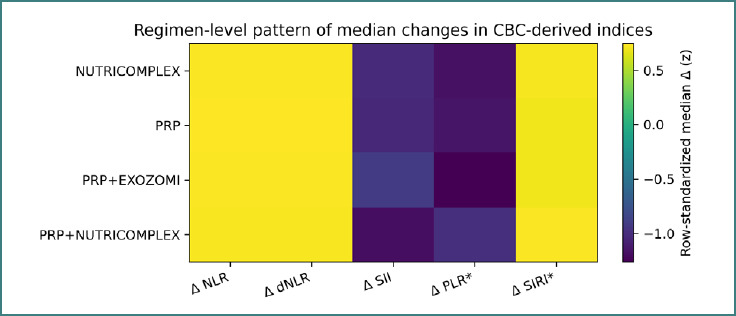
Heatmap of regimen-level median changes (Δ) in CBC-derived inflammatory indices. Values are row-standardized (z-score within each regimen) for visualization of relative patterns.

The distribution of slopes suggests that the immune-modulatory context also changed heterogeneously over follow-up. To summarize CBC-derived inflammatory index trajectories at the regimen level, we computed the median change (Δ) for each index within each regimen and visualized the resulting pattern as a heatmap (Figure 11).

The row-standardized heatmap emphasizes comparative patterning across indices within each regimen rather than absolute between-regimen ranking.

Associations between biomarker trajectories and clinical improvement were explored using Spearman correlations between %Δ total hair density and biomarker change scores (Δ), with FDR correction across the biomarker panel (Table 4).

**Table 4:** Spearman correlations between %Δ total hair density and biomarker change-scores (Δ)

Variable (Δ)	Spearman ρ	*N*	*P*	q (FDR)
Δ SIRI*	0.07	129	0.447	0.575
Δ dNLR	-0.02	129	0.851	0.957
Δ TGF-β1	0.00	129	0.961	0.961
Δ NLR	0.34	129	<0.001	<0.001
Δ PLR*	0.44	129	<0.001	<0.001
Δ SII	0.49	129	<0.001	<0.001
Δ TNF-α	-0.71	129	<0.001	<0.001
Δ VEGF	0.62	129	<0.001	<0.001
Δ Vitamin D	0.51	129	<0.001	<0.001

q-values are FDR-adjusted

The strongest FDR-ranked associations included Δ TNF-α (ρ = −0.71, *P* < 0.001, q < 0.001), Δ VEGF (ρ = 0.62, *P* < 0.001, q < 0.001), and Δ SII (ρ = 0.49, *P* < 0.001, q < 0.001). These findings should be interpreted as exploratory longitudinal co-movements rather than as evidence of causality.

## DISCUSSION

### Principal findings

In this ambispective real-world cohort, total hair density increased between baseline (T1) and follow-up (T2) on standardized trichoscopic assessment, with substantial inter-individual variability in both the magnitude and direction of change. Objective, software-assisted trichoscopy/phototrichogram approaches are well suited to detect such longitudinal shifts because they quantify density at the same scalp sites over time rather than relying on purely subjective clinical impressions [14].

### Trichoscopic improvement in the context of PRP evidence

Our trichoscopic findings align with prior clinical studies reporting improvements in hair parameters after PRP, including randomized, evaluator-blinded half-head designs and prospective clinical studies with follow-up beyond the early post-treatment period [14,15]. Evidence synthesis also supports a positive signal for PRP in androgenetic alopecia while emphasizing that outcomes remain heterogeneous across studies [4,16]. A major driver of this heterogeneity is protocol variability (including preparation systems, platelet concentration, activation strategy, injection technique, number of sessions, and follow-up intervals), which complicates direct cross-study comparisons and is consistent with the dispersion of %Δ responses observed in routine practice cohorts [4,6,16].

### Biological plausibility and rationale for a longitudinal multi-marker perspective

Mechanistic reviews describe PRP as biologically plausible for hair restoration because platelet concentrates deliver a coordinated set of growth factors and signaling mediators that may influence angiogenesis, cellular proliferation, and tissue remodeling relevant to follicular cycling [6]. Platelets are also a recognized circulating source of growth factors, including TGF-β family members, supporting the rationale for tracking growth-factor–related markers (e.g., VEGF, TGF-β1) when available [8]. Importantly, our approach prioritized within-subject trajectories (T2–T1) and a panel perspective rather than attributing response to a single baseline biomarker, thereby reflecting the multifactorial and heterogeneous nature of treatment response in routine clinical practice.

### Combination regimens: PRP with exosomes and PRP with nutricomplex

A practical strength of this cohort is the inclusion of real-world combination strategies. For PRP + exosome regimens, reviews and translational syntheses highlight a promising biological rationale (including intercellular signaling and pro-regenerative and immunomodulatory effects), but also emphasize major variability in product sources, characterization, dosing, and delivery, as well as the need for more rigorous comparative evidence [17–19]. Prospective clinical work combining exosomes with microneedling has reported improvements in hair density and patient-reported outcomes, but available studies remain relatively small and protocols heterogeneous [18]. Editorial and practice-oriented discussions also stress that commercialization has outpaced standardization, thereby reinforcing the need for cautious interpretation and stronger evidence standards in this area [20].

For nutraceutical/nutricomplex approaches, randomized placebo-controlled trial evidence exists for selected formulations and has demonstrated improvements in hair growth and quality outcomes in men with thinning hair [21]. Nevertheless, evidence synthesis across supplement categories remains heterogeneous, and network meta-analytic frameworks underscore that relative rankings are sensitive to formulation differences, outcome selection, and trial design [22,23]. Accordingly, regimen-level differences in %Δ density in our cohort should be interpreted as hypothesis-generating and potentially influenced by baseline severity, adherence, co-interventions, and confounding by indication.

### “Inflammatory status” as a pragmatic panel, not a single biomarker

We evaluated systemic inflammatory status using CBC-derived indices—NLR, dNLR, SII, PLR*, and SIRI*—together with soluble markers (vitamin D, TNF-α, VEGF, TGF-β1). A panel approach is pragmatic because each index weights leukocyte and platelet compartments differently and can capture partially distinct aspects of inflammatory balance. Large observational work in inflammatory alopecia contexts (e.g., alopecia areata) supports the broader concept that blood-derived ratios can reflect clinically relevant immune activity [9]. In addition, broader immunology-focused evidence indicates that indices such as SII are increasingly used as accessible markers across immune-mediated disease settings, supporting their feasibility as comparative metrics when used and reported consistently [24]. Recent longitudinal data in severe alopecia areata also suggest that systemic inflammatory indices can change over time alongside clinical improvement, supporting a trajectory-based interpretation rather than reliance on a single timepoint [10].

### Vitamin D and soluble markers in a longitudinal context

Vitamin D status has been repeatedly linked to alopecia disorders. A recent systematic review/meta-analysis across scarring and non-scarring alopecias reported a higher prevalence of vitamin D deficiency and lower vitamin D levels across several alopecia categories [13]. A separate meta-analysis focusing on non-scarring alopecia similarly reported reduced serum 25(OH)D levels and increased odds of deficiency [25]. In real-world cohorts, vitamin D trajectories can also reflect supplementation and seasonality; therefore, interpretation benefits from embedding vitamin D within a broader clinical and biomarker panel.

Soluble markers such as TNF-α and growth-factor–related markers (VEGF, TGF-β1) provide complementary information on inflammatory signaling and regenerative pathways, which are often discussed in PRP biology [6,8]. The heterogeneity observed in individual biomarker trajectories mirrors that in clinical response and supports the value of paired and distributional reporting rather than relying solely on group means.

### Linking biomarker trajectories to clinical improvement and multiplicity control

Our correlation analyses were exploratory and hypothesis-generating, aiming to identify biomarker change-scores (Δ) that track with clinical improvement (%Δ density). Because multiple biomarkers were evaluated, false discovery rate control was applied to reduce false positives in multi-marker screening [26]. The observed pattern supports a trajectory-based perspective: changes over time across multiple markers may better contextualize response heterogeneity than cross-sectional snapshots.

### Strengths and limitations

Strengths include objective trichoscopic quantification at two timepoints, longitudinal tracking of a pragmatic systemic biomarker panel, and inclusion of real-world regimen heterogeneity, including combination strategies used in routine care.

Limitations include the observational, nonrandomized design and potential confounding by indication across regimen groups. Follow-up timing reflects routine practice rather than a fixed protocol, and not all biomarkers were available for all participants at both timepoints. Soluble markers may be influenced by intercurrent conditions, supplementation, and laboratory variability. Correlation analyses do not establish causality and should be interpreted as exploratory.

### Implications and future directions

These findings support objective trichoscopy as a practical tool for monitoring response and highlight that response distributions are heterogeneous across individuals and regimens. From a biomarker standpoint, tracking NLR, dNLR, SII, PLR*, and SIRI* alongside selected soluble markers may provide a structured framework to describe systemic context during follow-up and guide hypothesis generation for future prospective work. Finally, because SIRI and related indices are increasingly applied across clinical outcomes research, clearer reporting of these indices in hair-loss cohorts may improve interpretability and comparability in future studies. [27]

## CONCLUSION

This complementary longitudinal analysis of a real-world PRP-based alopecia cohort integrates objective trichoscopic change with paired systemic biomarker trajectories. Hair density improved from baseline to follow-up, while CBC-derived inflammatory indices and soluble markers showed heterogeneous modulation over time. Framed cautiously and exploratorily, these results are consistent with the view that response heterogeneity is better understood as a multi-marker longitudinal process than as a single endpoint alone.
